# Comparative Physiology and Transcriptome Analysis of Young Spikes in Response to Late Spring Coldness in Wheat (*Triticum aestivum* L.)

**DOI:** 10.3389/fpls.2022.811884

**Published:** 2022-02-03

**Authors:** Gang Jiang, Muhammad A. Hassan, Noor Muhammad, Muhammad Arshad, Xiang Chen, Yonghan Xu, Hui Xu, Qianqian Ni, Binbin Liu, Wenkang Yang, Jincai Li

**Affiliations:** ^1^School of Agronomy, Anhui Agricultural University, Hefei, China; ^2^Agronomy Forage Production Section, Ayub Agricultural Research Institute, Faisalabad, Pakistan; ^3^Agriculture Department, Crop Reporting Service, Nankana Sahib, Pakistan; ^4^Jiangsu Collaborative Innovation Centre for Modern Crop Production, Nanjing, China

**Keywords:** anther connective tissue formation phase (ACFP), differentially expressed genes (DEGs), late spring coldness (LSC), physiology and transcriptome, *Triticum aestivum* L.

## Abstract

Late spring coldness (LSC) is critical for wheat growth and development in the Huang-Huai valleys of China. However, little is known about the molecular mechanisms for young spikes responding to low temperature (LT) stress during anther connective tissue formation phase (ACFP). To elucidate the molecular mechanisms associated with low temperature, we performed a comparative transcriptome analysis of wheat cultivars Xinmai26 (XM26: cold-sensitive) and Yannong19 (YN19: cold-tolerant) using RNA-seq data. Over 4000 differently expressed genes (DEGs) were identified under low temperature conditions (T1: 4°C) and freezing conditions (T2: −4°C) compared with control (CK: 16°C). The number of DEGs associated with two cultivars at two low temperature treatments (T1: 4°C and T2: −4°C) were 834, 1,353, 231, and 1,882 in four comparison groups (Xinmai26-CK vs. Xinmai26-T1, Xinmai26-CK vs. Xinmai26-T2, Yannong19-CK vs. Yannong19-T1, and Yannong19-CK vs. Yannong19-T2), respectively. Furthermore, to validate the accuracy of RNA-seq, 16 DEGs were analyzed using quantitative real-time RT-PCR. Several transcriptome changes were observed through Kyoto Encyclopedia of Genes and Genomes (KEGG) pathway functional enrichment analysis in plant hormone signal transduction, circadian rhythm-plant, and starch and sucrose metabolism under low temperature. In addition, 126 transcription factors (TFs), including *AP2-ERF, bHLH, WRKY, MYB, HSF*, and members of the *bZIP* family, were considered as cold-responsive. It is the first study to investigate DEGs associated with low temperature stress at the transcriptome level in two wheat cultivars with different cold resistance capacities. Most likely, the variations in transcription factors (TFs) regulation, and starch and sucrose metabolism contribute to different cold resistance capacities in the two cultivars. Further, physiological activities of superoxide dismutase (SOD), peroxidase (POD), catalase (CAT) enzymes, malondialdehyde (MDA), soluble sugar (SS), and sucrose contents were evaluated to investigate the negative impacts of low temperature in both cultivars. These findings provide new insight into the molecular mechanisms of plant responses to low temperature and potential candidate genes that required for improving wheat’s capacity to withstand low temperature stress.

## Introduction

Recent climate changes are incredibly detrimental to field crops and pose a severe threat to global food security (Hansen et al., 2012; Food and Agriculture Organization [FAO], 2020). Temperature extremes frequently occurred in the past decades (Masson-Delmotte et al., 2021). Particularly, extreme cold conditions frequently occur across the globe with varying intensity and duration (Barton et al., 2014). Low temperature (LT) stress limits active plant growth by causing mechanical injury and disturbing metabolic activity through crystallizing (Yadav, 2010). Wheat (*Triticum aestivum* L.) is a leading cereal in fulfilling global food needs (Hassan et al., 2021). LTs often hamper its growth and yield in the major wheat-growing regions of the world, such as China, the United States, Europe, and Australia (Holman et al., 2011; Trnka et al., 2014; Zheng et al., 2015b; Crimp et al., 2016; Xiao et al., 2018). In the meantime, the rise in global warming accelerated the wheat growth cycle and increased the risk of cold injury (Kodra et al., 2011; Augspurger, 2013; Zheng et al., 2015a; Li et al., 2016). Cold conditions also restrict active root water uptake, resulting in water deficiency in stem and triggers drought stress (Aroca et al., 2012), which instigates the inevitable damage to wheat growth. In recent years, the Huang-Huai wheat growing area of China often suffered from sudden late spring coldness (LSC) that disrupted the normal growth and lowered the final wheat yield. Frequent occurrence of LSC in this region causes a substantial reduction in final grain yield by 30–50% in severe cases, affecting nearly 42% of wheat sown areas (Zhang X. et al., 2011; Ji et al., 2017).

The damaging events of LSC on wheat usually occur between late March (jointing) and late April (booting) during sensitive reproductive growth phases; this adversely affect the yield as well as quality of wheat (Reinheimer et al., 2004). Wheat capacity to endure cold stress varies at different growth phases; during the early vegetative growth phase, it has shown efficient acclimation responses and effectively survived the cold period. In contrast, the reproductive phase is more vulnerable to cold stress, which causes a severe reduction in final wheat production (Thakur et al., 2010; Zinn et al., 2010), especially when LT coincides with other meteorological disasters (Zhang X. et al., 2011; Ji et al., 2017). Cromey et al. (1998) established that sensitivity to LT varies at different developmental stages of young spikes. The cold tolerance mechanism at the sensitive phase of spike development is not well explored (Zhong et al., 2008). During late spring, wheat plants are in the anther connective tissue formation phase (ACFP) and the tetrad development phase. The frost occurrence at this stage can cause substantial damage, such as floret and spike death, and impaired grain development. When ACFP began, young spikes showed disproportionally low resistance to cold and became more vulnerable (Zhong et al., 2008).

The inducer of CBF expression-C repeat binding factor-cold responsive (*ICE-CBF-COR*) pathway is a universal pathway related to cold stress tolerance in crop species (Guo et al., 2019). It is accomplished through various transcriptional and physiological processes, such as activation of cold regulated gene expressions (Zhu et al., 2007; Majláth et al., 2012), downstream regulation of photosynthesis, accumulation of osmoprotectants, and stimulation of antioxidant system (Theocharis et al., 2012). In addition, this trehalose (glucose disaccharide) is reported as a component of starch accumulation and a cold tolerance regulator (Fernandez et al., 2010).

Cold tolerant gene expressions play a vital role in plants’ counter-response to cold stress (Ganeshan et al., 2008). There are various cold-regulated genes (DHN: Dehydrins, LEA: Late-embryogenesis abundance, COR: Cold-responsive, ABR: Abscisic Acid Responsive) discovered in wheat (Guo et al., 2019). Transcription factors (TFs) are involved in the up/downstream regulation of gene expression. The TFs for LT genes regulation include *CBF/DREB* (dehydration-responsive element-binding) (Shinozaki and Yamaguchi-Shinozaki, 2000), *ICE* (Chinnusamy et al., 2003), *bZIP* (Takumi et al., 2003) *WRKY* (Qiu and Yu, 2009), and *MYB* (Mao et al., 2012). These TFs regulate the cold-induced gene expressions (i.e., *COR*, *PIPs*, and *TIPs*), and some of them also play a vital role in combating other abiotic stress, i.e., drought and salinity (Xue et al., 2006; Xu et al., 2008). These gene expressions induce various physiological and biochemical adjustments in the plant cell and gradually assist the plant in acclimatizing the LT conditions. The natural evolution of wheat plants has shown an ideal responsive mechanism to LT stress during harsh winters. It is a complex mechanism as cold-responsive (COR) gene expressions are entangled with several biochemical processes; any deviation from optimum temperature influences the active fluidity of the plant cell (Zheng et al., 2011). It has been stated that expressions of cold-responsive genes can be accelerated or inhibited by altering membrane fluidity (Örvar et al., 2000; Sangwan et al., 2001; Vaultier et al., 2006). Currently, over 450 cold-tolerant genes have been identified in wheat, but most of them were explored under acclimation responses and rarely reported in countering late spring coldness (Monroy et al., 2007; Winfield et al., 2010).

RNA sequencing (RNA-seq) is a popular and widely used method and was employed in studies on various plant species exposed to cold, such as apples, cabbage, maize, and tea (Lu et al., 2017; Li et al., 2019; Song et al., 2019; He et al., 2020). It was used in several studies about wheat genome responses to LT stress, but few have evaluated wheat transcriptomes under LT stress in spring (Kang et al., 2013; Zhang et al., 2014; Song et al., 2017; Díaz et al., 2019). In this experiment, we used two cultivars, Yannong19 (YN19: cold-tolerant) and Xinmai26 (XM26: cold-sensitive), as experimental materials to study their responses to LT stress in spring. We investigated the response of young spikes to LSC at a molecular level using transcriptome sequencing. Despite other studies describing transcriptomes in young spikes, the unique aspect of our research is using two wheat cultivars with varying sensitivity to LT and setting temperatures above (chilling) and below zero (freezing). The physiological indices of young spikes were investigated by evaluating the metabolic activities of superoxide dismutase (SOD), catalase (CAT), peroxidase (POD) enzymes, malondialdehyde (MDA) contents, soluble sugars (SS), and sucrose contents.

It is well known that plant signaling responses vary when temperatures drop suddenly or gradually (Kidokoro et al., 2017). Winfield et al. (2010) also established that cold responsive genes obtained from sudden cold shock were different than those identified in gradual temperature decline. Though the LT treatments were applied in growth chambers, care was taken to ensure that the pots would not be suddenly exposed to cold shock by shifting from field conditions to already set LT treatments. To better acclimate to the natural conditions of LT, the growth incubator temperature gradually declined from field temperature (16°C) to target LT treatments (4°C, −4°C) in 5 h duration, then subjected to LT treatments for next 4 h duration. Besides providing effective gene resources for wheat breeding, this study provided valuable insight into the molecular and genetics mechanisms involved in wheat resistance to LSC.

## Materials and Methods

### Plant Materials and Treatments

**FIGURE 1:**
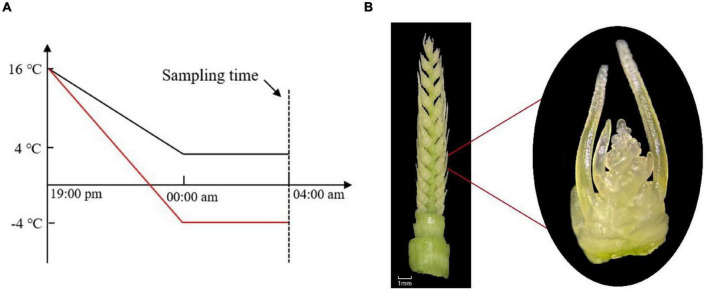
**(A)** Temperature gradually declined from field conditions (16°C) to target LT (4°C, −4°C) in 5 h, then in the next 4 h, were subjected to LT treatment. **(B)** Young spike of wheat at the anther connective tissue formation phase (ACFP).

This experiment was conducted in 2020–2021 at the Nongcuiyuan experimental station of Anhui Agricultural University (31.83°N, 117.24°E), Hefei, China. Two winter wheat cultivars with contrasting cold tolerance to LT, Yannong19 (YN19, cold-tolerant) and Xinmai26 (XM26, cold-sensitive), were used in the experimentation. Wheat cultivars were sown in pots of 26 cm diameter × 35 cm height, on November 1, 2020. There were 30 pots in total with three LT treatments [T1: 4°C, T2: −4°C, T3 (CK): 16°C], and five replications for each cultivar. Each pot was filled with 8 kg soil and 4.32 g compound fertilizer (N:P:K = 15:15:15) incorporated in it. The soil was taken from the field of 0∼20 cm upper plowing layer (having pH 6.5, organic matter 16.3 g⋅kg^–1^, available nitrogen 112.2 g⋅kg^–1^, available phosphorus 23.0 mg⋅g⋅kg^–1^, available potassium 161.6 mg⋅kg^–1^). Eight seedlings were maintained in each pot after emergence. All the pots were placed in the field conditions until they reached ACFP. We observed different growth stages and growth patterns of wheat plants under a microscope (OLYMPUS SZ2-ILST; Philippines; Tokyo, Japan). Moreover, at the ACFP phase, all the pots, except control treatments, were shifted to an ultra-low temperature incubator (DGXM-1008, Ningbo Jiangnan Instrument Factory). To better understand the natural conditions of LT variations, incubator temperature was gradually declined from field temperature (16°C) to target LT (4°C, −4°C) in 5 h duration, then subjected to LT treatments for next 4 h (Humidity: 75%, Light intensity: 0 μmolm^–2^s^–1^) (Figure 1A). Light intensity was adjusted to 0 μmol m^–2^s^–1^ as in the field conditions of Huang-Huai plains wheat plants suffered from LSC after midnight (Hassan et al., 2021). After LT treatments were finished, samples of spikes were collected from each pot and then, without any delay, frozen in liquid nitrogen and kept at −80°C for subsequent RNA extraction; three biological replicates were performed for each treatment having five technical replicates.

### Determination of Physiological Indexes of Young Spikes Under Cold Stress

The enzymatic activities of SOD, POD, CAT, and MDA contents in young spikes were determined following (Tan et al., 2008). The SS contents were assessed by the anthrone colorimetry method (Yemm and Willis, 1954). The sucrose contents were determined according to Zhang et al. (2019).

### Total RNA-Extraction and mRNA Library Construction

The total RNA from the spike tissues was extracted using the TRIzol kit (Invitrogen, Carlsbad, CA, United States). RNA was examined for quality and quantified using agarose gel and the NanoDrop 2500 (Thermo Fisher Scientific, United States). The Agilent 2100 was used for detecting the purity and integrity of RNA. Magnetized beads with Oligo (dT) attachment were used for mRNA purification. Thereafter, purified mRNA samples were subjected to RNA fragmentation. Afterward, the first strand cDNA was generated using random hexamer-directed reverse transcription, followed by synthesis of second strand cDNA. Incubation was conducted for end repair by adding RNA Index Adapters and A-Tailing Mix. As a result of the previous step, amplification of the cDNA fragments was carried out by PCR and Ampure XP beads were used for purification, followed by dissolution in EB solution. As a quality control measure validation was carried out on the Agilent Technologies 2100 Bioanalyzer. As a result of the previous step, the double stranded PCR products were heated, denatured, and circularized with the splint oligo sequencing for creation of final library. Then final library was configured using single-strand circle DNA (ssCir DNA). We amplified the final library with phi29 to make a DNA nano-ball (DNB), which contained more than 300 copies of each molecule. On the DNBSEQ-T7 platform, DNBs were loaded into the nano-array, and 150-base pair-end reads were generated. The data generated is then subjected to further analysis. Library construction and RNA sequencing were performed by TSINGKE Biotechnology Company (Nanjing, China).

### Quality Control, Mapping of RNA Sequencing Reads, and Genes Annotation

We first processed FASTAQ raw data (raw reads) using In House Perl scripts. Following that we removed ploy-N, adapters, and low quality reads from raw data to get clean reads (or clean data). By using the clean data, Q20, Q30, GC-content, and sequence duplication were calculated. High-quality, clean dataset was used for all downstream analyses. The clean reads were mapped to genome reference sequences^1^. Reads matching the reference genome were further analyzed and annotated. HISAT2 [(v2.2.0)]^2^ software tools were used to map with reference genome (Kim et al., 2015). Bowtie2 [(v2.2.5)]^3^ (Langmead and Salzberg, 2012) and StringTie [(v2.1.2)]^4^ (Pertea et al., 2015) softwares were used to align the clean reads and calculate gene expression profiles, respectively.

The quantification of gene expression levels was estimated based on Fragments Per Kilobase of Transcript Per Million Fragments Mapped (FPKM). We performed differentiation of expression analysis using DESeq2 [(v1.26.0)]^5^ (Love et al., 2014), that provides statistical routines for identifying differential expression in digital gene expression dataset by employing a model that was based on the negative binomial distribution. Benjamini and Hochberg’s method of controlling false discovery rate was then applied to adjust the resulting *P*-values. Genes with FDR < 0.05 and | log_2_(foldchange)| ≥ 1 found by DESeq2 were classified as differentially expressed. Gene functions were annotated using the following databases: COG (the database of clusters of orthologous groups of proteins)^6^, Pfam (the database of homologous protein family)^7^, Nr (NCBI non-redundant protein sequence database)^8^, eggNOG (Evolutionary Genealogy of Genes: Non-supervised Orthologous groups)^9^, KOG (the database of clusters of protein homology)^10^, KEGG (the database of Kyoto Encyclopedia of Genes and Genomes)^11^, Swiss-Prot (a manually annotated, non-redundant protein sequence database)^12^, and GO (gene ontology)^13^ annotation analysis.

### Quantitative Real-Time PCR Analysis

The qRT-PCR was performed to validate differential gene expressions in RNA-seq data. We extracted RNA from six distinct treatments (XMCK, XMT1, XMT2, YNCK, YNT1, and YNT2). The first cDNA was synthesized using The NovoScript^®^ Plus All-in-one 1st Strand cDNA Synthesis Super-Mix (Novoprotein, Suzhou, China), and qRT-PCR was performed using 2X M5 HiPer SYBR Premix EsTaq with Tli RNaseH (Mei5bio, Beijing, China); in the CFX Connect real-time PCR detection system according to instructions. The 25 μl reaction system includes 12.5 μl of 2x M5 HiPer SYBR Premix EsTaq, 0.5 μl each of 10 μM primers, 2 μl of diluted cDNA, and 9.5 μl ddH_2_O. The standard PCR amplification procedure had pre-denaturation at 95°C for 2 min, followed by 39 cycles of 5 s at 95°C, 30 s at 60°C. The primers were designed on NCBI and sent to Sangon Biotech Company (Shanghai, China) for synthesis. The relative expression levels of DEGs were normalized as the expression of *TaActin*, which was used as endogenous control and calculated by the 2^–ΔΔCt^ method (Livak and Schmittgen, 2001). The primers (forward and reverse) sequences and reference gene *TaActin* are mentioned in Supplementary Table 1.

### Statistical Analysis

Statistical analysis of physiological data of young spikes were computed in IBM SPSS v24.0 (IBM Corp., Armonk, NY, United States). Duncan’s multiple range test was performed to determine the statistically significant differences. Comparison of treatment means was based on least significant difference (LSD) at a 0.05 probability level. Venn diagram was made by online software^14^. The clustering heat map (CHM) was constructed by TB tools (Chen et al., 2020). Graphical presentations were made on the Microsoft Excel (2016) program (Microsoft Corporation, United States).

## Results

### Physiological Response Patterns Under Different Cold Treatment

Wheat young spikes at ACFP (Figure 1B) were highly vulnerable to LT stress. Physiological analyses were conducted on young spikes at the ACFP immediately after being exposed to cold stress.

#### Antioxidant Enzymes

**FIGURE 2:**
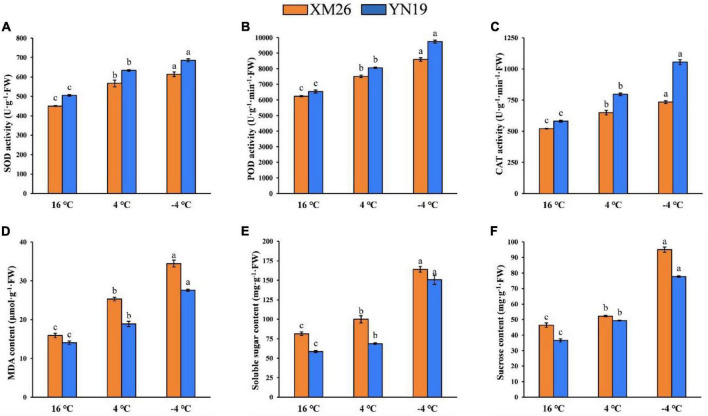
Physiological indices of Xinmai26 (XM26) and Yannong19 (YN19) under cold stress; **(A)** Superoxide dismutase (SOD) activity, **(B)** Peroxidase (POD) activity, **(C)** Catalase (CAT) activity, **(D)** malondialdehyde (MDA) content, **(E)** Soluble sugar content, and **(F)** Sucrose content. Data mean ± SD (*n* = 3). Different lowercase letters on the columns indicate significant differences between temperature treatments (*P* ≤ 0.05).

The activities of antioxidant enzymes (SOD, POD, and CAT) in young spikes were significantly different under varying LT treatments (Figures 2A–C). Both cultivars, XM26 and YN19, were exhibited increased activity of SOD by 26 and 29%, POD by 20 and 23%, CAT by 25 and 37% at 4°C, respectively, as compared to control (16°C). Similarly, XM26 and YN19 exhibited increased activity of SOD by 36 and 43%, POD by 23 and 49%, CAT by 41 and 82% at −4°C, respectively, as compared to control (16°C).

#### Malondialdehyde

The activity of MDA was also increased under both LT treatments, indicating an elevated intensity of lipids peroxidation (Figure 2D). Both cultivars, XM26 and YN19, were exhibited that MDA contents were increased by 59 and 34% at 4°C, respectively, as compared to control (16°C). Similarly, XM26 and YN19 exhibited that MDA content were increased by 116 and 96% at −4°C, respectively, compared to the control (16°C). Eventually, young wheat spikes were severely damaged by low temperatures, and further temperature decline increased the damage.

#### Soluble Sugars and Sucrose

Soluble sugars (SS), which act as osmoregulatory substances, reduce membrane damage, and the content of SS and sucrose increases as temperature decreases (Figures 2E,F). The SS contents in both cultivars, XM26 and YN19, were increased by 23 and 17% at 4°C, respectively, as compared to control (16°C). Similarly, the SS contents in XM26 and YN19 were increased by 102 and 157% at −4°C, respectively, as compared to control (16°C). In the same way, sucrose contents (as an integral component of SS) in XM26 and YN19 were increased by 13 and 35% at 4°C, respectively, as compared to control (16°C). Likewise, at −4°C, XM26 and YN19 were shown increased sucrose contents by 105 and 112%, respectively, as compared to control (16°C).

The physiological data analysis of SOD, POD, CAT, MDA, SS, and sucrose contents demonstrated significant difference (*p* ≤ 0.05) under two LT treatments (4°C, −4°C), compared with the control (16°C).

### RNA Sequencing Analysis of Young Spikes of YN19 and XM26 Under Low Temperature Stress

We obtained 18 library reads, including six different treatments (XMCK, XMT1, XMT2, YNCK, YNT1, and YNT2), and each group has three biological replicates. In total, 160.55 GB of raw data were obtained. The number of clean paired end reads in each sample ranged from 18,194,227∼39,996,772. The clean reads of each sample was aligned to reference genome sequence (see text footnote 1), their comparison efficiency ranged from 94.48 to 96.35%. The number of reads that matched the unique position in the reference genome ranged from 89.26 to 91.56%, and the number of reads that matched to multiple positions of the reference genome ranged from 4.59 to 6.31% (Table 1). The GC contents ranged from 49.46 to 50.97%, and the average percentage of Q30 was above 90.89% (Table 2). All three biological replicates had high Pearson’s correlation coefficients (*R*^2^ = 0.82∼0.98) (Supplementary Table 2). The results indicated that the sequencing data met the quality requirements for subsequent analysis to a greater extent. These reads are publicly available in the SRA database of NCBI with the Bio-project accession number PRJNA770017.

**TABLE 1:** Summary of RNA sequencing data and mapped reads to the reference genome (ftp://ftp.ensemblgenomes.org/pub/plants/release-37/fasta/triticum_aestivum/dna/Triticum_aestivum.TGACv1.dna.toplevel.fa.gz).

ID	Total Reads	Mapped Reads	Uniquely Mapped Reads	Multiple Map Reads	Reads Map to “+”	Reads Map to “−”
XMCK-1	74409288	71,231,043 (95.73%)	67,382,415 (90.56%)	3,848,628 (5.17%)	34,812,501 (46.79%)	34,728,291 (46.67%)
XMCK-2	64851748	62,296,887 (96.06%)	59,171,042 (91.24%)	3,125,845 (4.82%)	30,514,404 (47.05%)	30,464,442 (46.98%)
XMCK-3	64111278	61,720,589 (96.27%)	58,563,290 (91.35%)	3,157,299 (4.92%)	30,244,333 (47.17%)	30,171,740 (47.06%)
XMT1-1	79993544	77,047,606 (96.32%)	72,916,188 (91.15%)	4,131,418 (5.16%)	37,705,127 (47.14%)	37,582,408 (46.98%)
XMT1-2	64068134	61,707,839 (96.32%)	58,544,457 (91.38%)	3,163,382 (4.94%)	30,230,645 (47.19%)	30,139,709 (47.04%)
XMT1-3	68380744	65,816,453 (96.25%)	62,398,861 (91.25%)	3,417,592 (5.00%)	32,224,263 (47.12%)	32,116,132 (46.97%)
XMT2-1	48785110	46,903,584 (96.14%)	44,666,249 (91.56%)	2,237,335 (4.59%)	22,998,465 (47.14%)	22,921,172 (46.98%)
XMT2-2	66650990	64,218,416 (96.35%)	60,010,401 (90.04%)	4,208,015 (6.31%)	31,162,409 (46.75%)	30,410,987 (45.63%)
XMT2-3	54788160	52,781,522 (96.34%)	50,010,353 (91.28%)	2,771,169 (5.06%)	25,834,908 (47.15%)	25,728,318 (46.96%)
YNCK-1	57915092	55,331,759 (95.54%)	52,415,579 (90.50%)	2,916,180 (5.04%)	27,057,599 (46.72%)	27,004,276 (46.63%)
YNCK-2	56056594	53,592,469 (95.60%)	50,779,125 (90.59%)	2,813,344 (5.02%)	26,216,937 (46.77%)	26,151,875 (46.65%)
YNCK-3	54090312	51,656,933 (95.50%)	49,061,382 (90.70%)	2,595,551 (4.80%)	25,289,942 (46.76%)	25,239,777 (46.66%)
YNT1-1	56465704	53,991,304 (95.62%)	51,135,696 (90.56%)	2,855,608 (5.06%)	26,409,269 (46.77%)	26,333,900 (46.64%)
YNT1-2	36388454	34,849,119 (95.77%)	33,043,584 (90.81%)	1,805,535 (4.96%)	17,048,145 (46.85%)	17,008,665 (46.74%)
YNT1-3	37058252	35,463,270 (95.70%)	33,589,282 (90.64%)	1,873,988 (5.06%)	17,321,957 (46.74%)	17,227,241 (46.49%)
YNT2-1	63992398	61,092,185 (95.47%)	58,000,131 (90.64%)	3,092,054 (4.83%)	29,888,564 (46.71%)	29,789,810 (46.55%)
YNT2-2	70675454	66,777,113 (94.48%)	63,083,247 (89.26%)	3,693,866 (5.23%)	32,531,208 (46.03%)	32,395,518 (45.84%)
YNT2-3	54602940	52,033,128 (95.29%)	49,381,547 (90.44%)	2,651,581 (4.86%)	25,426,370 (46.57%)	25,320,864 (46.37%)

*ID, sample analysis number; Total Reads: number of Clean Reads by the single end; Mapped Reads, number of Reads mapped to the reference genome and percentage of Clean Reads; Uniquely Mapped Reads, number of Reads mapped to the unique position of the reference genome and percentage of Clean Reads; Multiple Map Reads, number of Reads mapped to the multiple positions of the reference genome and percentage of Clean Reads; Reads Map to “+” described the number of reads to the forward strand of the reference genome and the percentage of Clean Reads; Reads Map to “−” described the number of reads to the reverse strand of the reference genome and the percentage of Clean Reads.*

*XMCK, Xinmai26 was treated at 16°C; XMT1, Xinmai26 was treated at 4°C for 4 h; XMT2, Xinmai26 was treated at −4°C for 4 h; YNCK, Yannong19 was treated at 16°C; YNT1, Yannong19 was treated at 4°C for 4 h; YNT2, Yannong19 was treated at −4°C for 4 h.*

**TABLE 2:** Statistical table of RNA-seq data.

Samples	Clean reads	Clean bases	GC Content	% ≥Q30
XMCK-1	37204644	11128072008	0.4971	0.9158
XMCK-2	32425874	9701400534	0.4974	0.9089
XMCK-3	32055639	9593928134	0.4946	0.918
XMT1-1	39996772	11972479266	0.4994	0.9274
XMT1-2	32034067	9589526096	0.498	0.9237
XMT1-3	34190372	10227654524	0.4986	0.9235
XMT2-1	24392555	7299008476	0.498	0.9101
XMT2-2	33325495	9972576102	0.5009	0.9205
XMT2-3	27394080	8197674646	0.4973	0.9245
YNCK-1	28957546	8665526544	0.499	0.9162
YNCK-2	28028297	8390154156	0.4992	0.9164
YNCK-3	27045156	8098482182	0.4946	0.91
YNT1-1	28232852	8449583814	0.4968	0.9156
YNT1-2	18194227	5444794420	0.4956	0.9171
YNT1-3	18529126	5546648882	0.4972	0.9164
YNT2-1	31996199	9576365178	0.5049	0.9125
YNT2-2	35337727	10536644946	0.5097	0.9167
YNT2-3	27301470	8160543218	0.5028	0.9134

*Samples, sample analysis number; Clean reads, total number of pair-end Reads in Clean Data; Clean bases, total number of bases in Clean Data; GC content, GC content of Clean Data, i.e., the percentage of G and C bases in Clean Data; GC content, GC content of Clean Data, i.e., the percentage of G and C bases in Clean Data out of the total bases; % ≥Q30, percentage of bases with Clean Data quality value greater than or equal to 30.*

*XMCK, Xinmai26 was treated at 16°C; XMT1, Xinmai26 was treated at 4°C for 4 h; XMT2, Xinmai26 was treated at −4°C for 4 h; YNCK, Yannong19 was treated at 16°C; YNT1, Yannong19 was treated at 4°C for 4 h; YNT2, Yannong19 was treated at −4°C for 4 h.*

### Differently Expressed Genes Obtained via Comparison of Two Cultivars in Different Treatments

**FIGURE 3:**
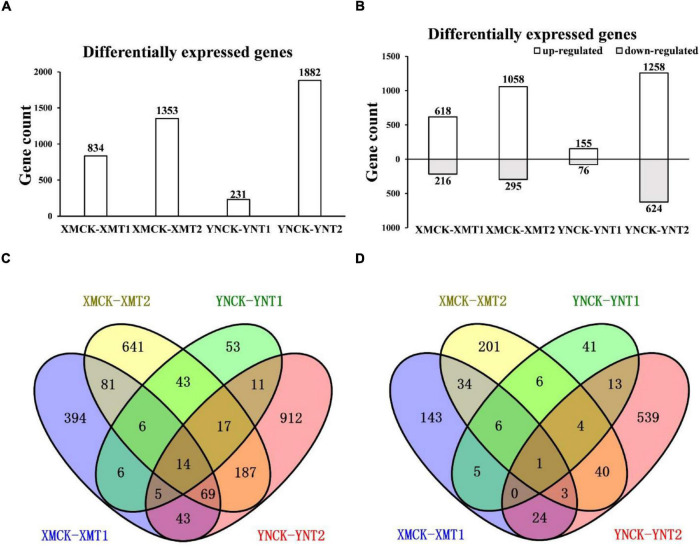
**(A)** The number of DEGs in each comparison group, **(B)** The number of upregulated and downregulated DEGs in each comparison group, **(C)** Venn diagram of upregulated DEGs in each comparison group, and **(D)** Venn diagram of downregulated DEGs in each comparison group. XMCK, Xinmai26 was treated at 16°C; XMT1, Xinmai26 was treated at 4°C for 4 h; XMT2, Xinmai26 was treated at −4°C for 4 h; YNCK, Yannong19 was treated at 16°C; YNT1, Yannong19 was treated at 4°C for 4 h; YNT2, Yannong19 was treated at −4°C for 4 h.

To understand the variations within the same cultivar under varying LT treatments and different cultivars with the same LT treatments, gene expression profiles of XM26 and YN19 were further analyzed by FPKM values (Supplementary Material). We obtained the DEGs of the single cultivar under different LT treatments and the DEGs of two cultivars under the same treatments through comparative analysis. A total of 231 (155 upregulated and 76 downregulated) and 834 (618 upregulated and 216 downregulated) DEGs were identified at 4°C, and 1,882 (1,258 upregulated and 624 downregulated) and 1,353 (1,058 upregulated and 295 downregulated) DEGs at −4°C in YN19 and XM26, respectively (Figures 3A,B). It is figured out that a cultivar responded differently to varying LT treatments, and different cultivars reacted differently to the same treatment. Among 3,089 upregulated genes, 14 were identified in all groups, and 170 and 47 genes were uniquely identified in XM26 and YN19, respectively; these genes involved in the response process of cold and freezing injury (Figure 3C). The number of shared genes identified in XM26 and YN19 were 31 (XMCK-XMT1 vs. YNCK-YNT1) and 287 (XMCK-XMT2 vs. YNCK-YNT2) at 4°C and −4°C, respectively. Among 1211 downregulated genes, only one gene was identified in all groups, while 44 and 18 genes were exclusively identified in XM26 and YN19, respectively, at two different LT patterns (Figure 3D). The large number of genes were identified only in specific comparison groups, indicating that the plant regulatory mechanisms significantly differ under two LT treatments. It also indicated that the cold tolerance mechanism of two cultivars also varied significantly.

### Functional Annotations and Enrichment Analysis of Differently Expressed Genes

**FIGURE 4:**
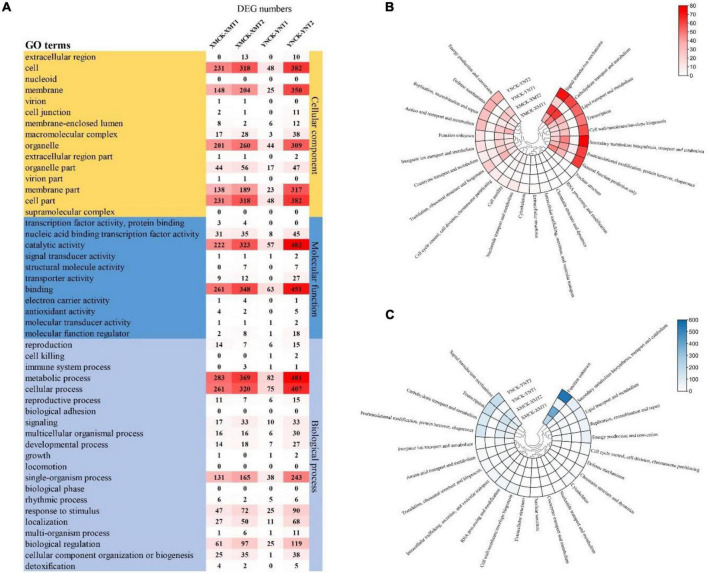
**(A)** The number of DEGs for GO terms in each comparison group, **(B)** Heat map of clustering of the number of DEGs annotated by COG database, and **(C)** Heat map of clustering of the number of DEGs annotated by eggNOG database. XMCK, Xinmai26 was treated at 16°C; XMT1, Xinmai26 was treated at 4°C for 4 h; XMT2, Xinmai26 was treated at −4°C for 4 h; YNCK, Yannong19 was treated at 16°C; YNT1, Yannong19 was treated at 4°C for 4 h; YNT2, Yannong19 was treated at −4°C for 4 h.

Gene expression profiles of the identified DEGs were compared with protein databases to determine each gene’s function. Functional annotation of DEGs made for each database (COG, GO, and eggNOG) shows the number of genes annotated to each DEG set (Table 3). The functional groups of DEGs in the GO database were categorized into three groups: cellular components (CC), molecular functions (MF), and biological processes (BP) (Figure 4A). Both cultivars, XM26 and YN19, showed a similar trend of functional enrichment. In accordance to their location and specific functionality, each functional group further distinguished on the basis of number of enriched DEGs, such as in CC most enriched pathways were found in cell (231, 318, 48, and 382 DEGs), membrane (148, 204, 25, and 350 DEGs) and organelle (201, 260, 44, and 309 DEGs); in MF most enriched pathways were found in catalytic activity (222, 323, 57, and 482 DEGs) and binding (261, 348, 63, and 451 DEGs); in BP most enriched pathways were found in metabolic process (283, 369, 82, and 481 DEGs), cellular process (261, 320, 75, and 407 DEGs), single-organism process (131, 165, 38, and 243 DEGs) and biological regulation (61, 97, 25, and 119 DEGs). The number of DEGs in each group was expressed according to the following comparison groups: XMCK-XMT1, XMCK-XMT2, YNCK-YNT1, and YNCK-YNT2, respectively. Both cultivars exhibited a similar trend, indicating that there are more pathways for plants to respond to freezing stress as more DEGs were detected under −4°C. In addition to the response to stimulus and transcription factor binding activity, other processes may play a key role in wheat’s ability to withstand cold temperatures.

**TABLE 3:** Summary of differentially expressed genes (DEGs) in different databases.

DEG Set	Total	COG	GO	KEGG	KOG	NR	Pfam	Swiss-Prot	eggNOG
XMCK-XMT1	813	331	517	305	380	751	657	631	794
XMCK-XMT2	1319	472	715	406	561	1194	983	972	1268
YNCK-YNT1	222	74	126	87	109	205	161	157	211
YNCK-YNT2	1828	636	999	586	817	1678	1413	1379	1760

*DEG Set, name of the differentially expressed gene set; Total, total number of DEGs annotated, and total number of DEGs annotated in different databases, i.e., COG, GO, KEGG, KOG, NR, Pfam, Swiss-Prot, and eggNOG.*

*XMCK, Xinmai26 was treated at 16°C; XMT1, Xinmai26 was treated at 4°C for 4 h; XMT2, Xinmai26 was treated at −4°C for 4 h; YNCK, Yannong19 was treated at 16°C; YNT1, Yannong19 was treated at 4°C for 4 h; YNT2, Yannong19 was treated at −4°C for 4 h.*

Gene ontology terms are represented on a directed acyclic graph (DAG), with more detail defined from top to bottom, and the darker the colors, the greater the enrichment significance (Supplementary Figures 1–3). The functional groups of CC such as photosystem (GO:0009521) and chloroplast thylakoid (GO:0009534), intracellular membrane-bounded organelles (GO:0043231) and plastid stroma (GO:0009532), nuclear lumen (GO:0031981) and intracellular (GO:0005622), plasma membrane part (GO:0044459) and protein acetyltransferase complex (GO:0031248) were significantly enriched in the following comparison groups of XMCK-XMT1 (Supplementary Figure 1A), XMCK-XMT2 (Supplementary Figure 1B), YNCK-YNT1 (Supplementary Figure 1C), YNCK-YNT2 (Supplementary Figure 1D), respectively. The functional groups of MF showed significant enrichment pathways in beta-fructofuranosidase activity (GO:0004564) and sigma factor activity (GO:0016987), beta-fructofuranosidase activity (GO:0004564) and pyrophosphatase activity (GO:0016462), oxidoreductase activity (in which oxidizing metal ions and oxygen as acceptor) (GO:0016724) and iron ion binding (GO:0005506), transferase activity (transferyl acyl groups) (GO:0016746) and UDP-glucosyltransferase activity (GO:0035251) in the following comparison groups XMCK-XMT1 (Supplementary Figure 2A), XMCK-XMT2 (Supplementary Figure 2B), YNCK-YNT1 (Supplementary Figure 2C), YNCK-YNT2 (Supplementary Figure 2D), respectively. The functional group BP significantly responded to temperature stimulus (GO:0009266) and reactive oxygen species (GO:0000302), transcription (DNA-templated) (GO:0006351) and Phosphorus metabolic process (GO:0006793), response to temperature stimulus (GO:0009266) and circadian rhythm (GO:0007623), response to reactive oxygen species (ROS) (GO:0000302) and response to temperature stimulus (GO:0009266) in the following comparison groups XMCK-XMT1 (Supplementary Figure 3A), XMCK-XMT2 (Supplementary Figure 3B), YNCK-YNT1 (Supplementary Figure 3C), YNCK-YNT2 (Supplementary Figure 3D), respectively. We found that the pathways of significant enrichment between the two cultivars differed significantly under different LT treatments, and the DAG gave us insight into the specific metabolic processes under LT conditions in plants.

Clusters of orthologous database was used to classify direct homologs of gene products (Figure 4B). We found a similar distribution between various comparison groups. Most DEGs were enriched in all comparison groups in carbohydrate transport and metabolism, signal transduction mechanisms, general function proteins, post-translational modification chaperones translation, and amino acid transport and metabolism. Similarly, most DEGs were also enriched in signal transduction mechanisms, carbohydrate transport and metabolism, transcription, post-translational modification, protein turnover, and chaperones based on the eggNOG database (Figure 4C). This demonstrates that signal transduction mechanisms, post-translational modification, protein turnover, chaperones, transcription, and carbohydrate transport and metabolism are important pathways in responding to low temperature treatments. The varying number of DEGs among cultivars concerning these pathways may be closely related to their ability to tolerate cold stress.

### Functional Kyoto Encyclopedia of Genes and Genomes Pathway Analysis Between Two Cultivars

Based on pathway enrichment analysis for specific DEGs in Kyoto Encyclopedia of Genes and Genomes (KEGG), we determined whether these two genotypes were involved in biological processes under cold stress. The pathways enriched by DEGs for each comparison group were different under 4°C and −4°C, respectively (Table 4). In XMCK-XMT1, the most enriched pathways for DEGs were plant pathogen interactions (ko04626 and 19 DEGs), circadian rhythm-plant (ko04712 and 18 DEGs), arginine and proline metabolism (ko00330 and 16 DEGs); in XMCK-XMT2, we identified biosynthesis of cofactors (ko01240 and 18 DEGs), plant hormone signal transduction (ko04075 and 15 DEGs), and phenylpropanoid biosynthesis (ko00940 and 15 DEGs). In YNCK-YNT1, photosynthesis-antenna proteins (ko00196 and 11 DEGs), circadian rhythm-plant (ko04712 and 10 DEGs), and biosynthesis of cofactors were found (ko01240 and 8 DEGs), and in YNCK-YNT2, plant-pathogen interaction (ko04626 and 35 DEGs), fatty acid elongation (ko00062 and 25 DEGs), phenylpropanoid biosynthesis (ko00940 and 23 DEGs) were enriched. These results suggest that plants respond to different metabolic pathways to acclimatize different levels of LT stress. However, some LT-induced shared pathways were found in different comparison groups, for instance plant hormone signal transduction, phenylpropanoid biosynthesis, and plant-pathogen interaction, acclimatizing to different LT regimes correlated to these pathways.

**TABLE 4:** The top 20 KEGG pathways with the largest number of differentially expressed genes in each comparison group.

XMCK-XMT1			XMCK-XMT2			YNCK-YNT1			YNCK-YNT2		
Pathway	ko_ID	DEGs	Pathway	ko_ID	DEGs	Pathway	ko_ID	DEGs	Pathway	ko_ID	DEGs
Plant-pathogen interaction	ko04626	19	Biosynthesis of cofactors	ko01240	18	Photosynthesis – antenna proteins	ko00196	11	Plant-pathogen interaction	ko04626	35
Circadian rhythm – plant	ko04712	18	Plant hormone signal transduction	ko04075	15	Circadian rhythm – plant	ko04712	10	Fatty acid elongation	ko00062	25
Arginine and proline metabolism	ko00330	16	Phenylpropanoid biosynthesis	ko00940	15	Biosynthesis of cofactors	ko01240	8	Phenylpropanoid biosynthesis	ko00940	23
Plant hormone signal transduction	ko04075	14	Amino sugar and nucleotide sugar metabolism	ko00520	14	Thiamine metabolism	ko00730	6	Glutathione metabolism	ko00480	22
Fatty acid elongation	ko00062	11	Plant-pathogen interaction	ko04626	14	Spliceosome	ko03040	4	Starch and sucrose metabolism	ko00500	21
Carbon metabolism	ko01200	11	Starch and sucrose metabolism	ko00500	14	Plant hormone signal transduction	ko04075	3	Circadian rhythm – plant	ko04712	21
Photosynthesis – antenna proteins	ko00196	10	Arginine and proline metabolism	ko00330	12	Arginine and proline metabolism	ko00330	3	Plant hormone signal transduction	ko04075	19
Glycerophospholipid metabolism	ko00564	9	Ribosome	ko03010	12	Cysteine and methionine metabolism	ko00270	3	Biosynthesis of cofactors	ko01240	18
Protein processing in endoplasmic reticulum	ko04141	9	MAPK signaling pathway – plant	ko04016	12	Alanine, aspartate and glutamate metabolism	ko00250	2	Cutin, suberin and wax biosynthesis	ko00073	17
Spliceosome	ko03040	8	Beta-Alanine metabolism	ko00410	11	Pyrimidine metabolism	ko00240	2	Amino sugar and nucleotide sugar metabolism	ko00520	15
Beta-Alanine metabolism	ko00410	8	Circadian rhythm – plant	ko04712	11	RNA degradation	ko03018	2	Photosynthesis – antenna proteins	ko00196	14
Phenylpropanoid biosynthesis	ko00940	8	Carbon metabolism	ko01200	10	Cyanoamino acid metabolism	ko00460	2	Photosynthesis	ko00195	14
Carbon fixation in photosynthetic organisms	ko00710	8	Oxidative phosphorylation	ko00190	10	Protein processing in endoplasmic reticulum	ko04141	1	Ascorbate and aldarate metabolism	ko00053	13
Biosynthesis of cofactors	ko01240	8	Thiamine metabolism	ko00730	9	DNA replication	ko03030	1	Oxidative phosphorylation	ko00190	12
Fructose and mannose metabolism	ko00051	6	Peroxisome	ko04146	9	Starch and sucrose metabolism	ko00500	1	Carbon metabolism	ko01200	12
Glutathione metabolism	ko00480	6	Ascorbate and aldarate metabolism	ko00053	9	Porphyrin and chlorophyll metabolism	ko00860	1	Arginine and proline metabolism	ko00330	12
Pentose phosphate pathway	ko00030	6	Ubiquinone and other terpenoid-quinone biosynthesis	ko00130	9	Peroxisome	ko04146	1	Glycerolipid metabolism	ko00561	11
Pantothenate and CoA biosynthesis	ko00770	6	Photosynthesis	ko00195	8	Endocytosis	ko04144	1	Beta-Alanine metabolism	ko00410	10
Biosynthesis of amino acids	ko01230	6	Fatty acid elongation	ko00062	7	Glycerolipid metabolism	ko00561	1	Fatty acid metabolism	ko01212	9
Glycolysis/Gluconeogenesis	ko00010	6	Spliceosome	ko03040	7	MAPK signaling pathway – plant	ko04016	1	Ubiquinone and other terpenoid-quinone biosynthesis	ko00130	9

*Pathways, KEGG pathway name; ko_ID, KEGG Orthology ID; DEGs, number of differentially expressed genes. XMCK, Xinmai26 was treated at 16°C; XMT1, Xinmai26 was treated at 4°C for 4 h; XMT2, Xinmai26 was treated at −4°C for 4 h; YNCK, Yannong19 was treated at 16°C; YNT1, Yannong19 was treated at 4°C for 4 h; YNT2, Yannong19 was treated at −4°C for 4 h.*

**FIGURE 5:**
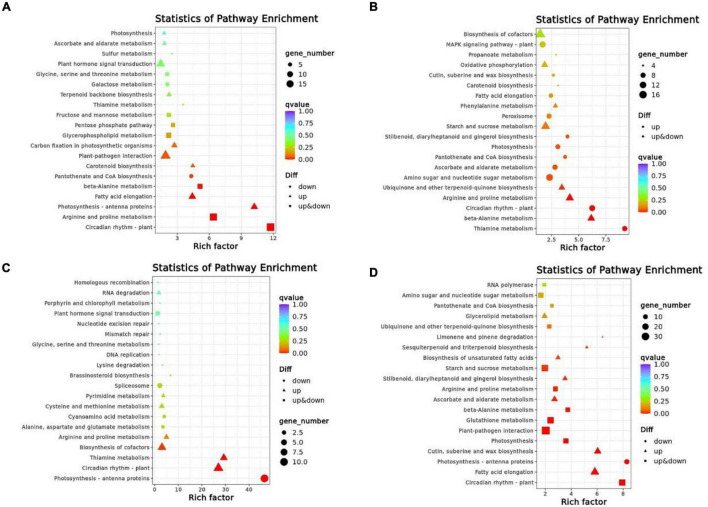
A scattered plot of the KEGG pathway for DEGs in each of the comparison group [**(A)** XMCK-XMT1, **(B)** XMCK-XMT2, **(C)** YNCK-YNT1, and **(D)** YNCK-YNT2]. Each circle in the figure represents a KEGG pathway. The ordinate represents the pathway name, and the horizontal axis represents enrichment factor, the ratio of the proportion of differential genes annotated to this pathway relative to the proportion of genes annotated to this pathway in all genes. Enrichment level of DEGs in this pathway is more significant when the enrichment factor is high. The color of the circle represents *q*-value, which was the *P*-value corrected by multiple hypothesis testing. DEGs in this pathway have better enrichment significance when the *q*-value is smaller. Each circle indicates the number of genes enriched in the pathway, and the larger the circle, the more genes. XMCK, Xinmai26 was treated at 16°C; XMT1, Xinmai26 was treated at 4°C for 4 h; XMT2, Xinmai26 was treated at −4°C for 4 h; YNCK, Yannong19 was treated at 16°C; YNT1, Yannong19 was treated at 4°C for 4 h; YNT2, Yannong19 was treated at −4°C for 4 h.

In order to determine if DEGs are significantly different in their pathways, we performed enrichment analyses within the top 20 KEGG pathways with the lowest significant *q*-values (Figure 5). In XMCK-XMT1, the most significant enrichment pathways were circadian rhythm-plant (ko04712), arginine and proline metabolism (ko00330), while in XMCK-XMT2, thiamine metabolism (ko00730), circadian rhythm-plant (ko04712), and beta-alanine metabolism (ko00410) pathways were significantly different. In YNCK-YNT1, the most significant enrichment pathways are photosynthesis-antenna proteins (ko00196), thiamine metabolism (ko00730), and circadian rhythm-plant (ko04712), while in YNCK-YNT2, phenylpropanoid biosynthesis (ko00940), circadian rhythm-plant (ko04712), cutin, suberin, and wax biosynthesis (ko00073) pathways were significantly different. These results indicated that the most significant enrichment pathways in the four comparison groups were considerably distinct, and they may play a vital role in the cold tolerance of wheat.

### Key Regulation Pathway Associated With Cold Stress

Annotations in the COG and eggNOG databases indicated that most of the DEGs were involved in (i) signal transduction, (ii) transcription, and (iii) carbohydrate transport. Primarily after sensing abiotic stress, calcium ions and phytohormones play a crucial role in signal transduction. Secondly, TFs, and starch and sucrose metabolism are vital components for transcription and carbohydrate transport, respectively. We then evaluated the expression of genes associated with them in each comparison group.

#### Ca^2+^ and Hormones Signal Transduction Differently Expressed Genes Associated With Cold Stress

We further analyzed DEGs related to calcium (Ca^2+^) and plant signal transduction through KEGG and Swiss-Prot protein databases. There were 26 DEGs identified in the Ca^2+^ signal transduction mechanism, among which both cultivars commonly shared eight DEGs, which were mainly associated with calcium-binding protein (Supplementary Table 3). Seven and 11 DEGs were uniquely identified in XM26 and YN19, respectively. The expression levels of seven DEGs identified in XM26 (TRIAE_CS42_1DS_TGACv1_080510_AA0249290; TRIAE_CS 42_6BL_TGACv1_499456_AA1582950 etc.), were upregulated at −4°C compared with 4°C. The expression levels of 11 DEGs identified in YN19 (TRIAE_CS42_1DS_TGACv1_080510_ AA0249290; TRIAE_CS42_6BL_TGACv1_499456_AA1582950 etc.) were upregulated at −4°C compared with 4°C. These genes are mainly related to calcium-binding protein, calcium-dependent protein kinase, and calcium uniporter protein, and highly expressed in response to the lower temperature.

There were 35 DEGs identified in both cultivars, which were involved in hormone signal transduction (Supplementary Table 4). Among which 7 DEGs were commonly shared in two cultivars, mainly related to auxin-responsive protein genes, whereas 13 and 15 DEGs were exclusively found in XM26 and YN19, respectively, indicating the common and cultivar-specific regulations under varying cold regimes. In this research, most of the DEGs were identified in ABA protein phosphatase 2C (PP2C), Auxin (*IAA12*, *IAA14*, and *IAA31*), and Ethylene (*EIL2* and *EBF1*) signaling pathways. Some DEG expressions were specific to cultivars involved in wheat cold tolerance, such as PP2C genes, which were uniquely identified in XM26. The expression of these genes may be one of the reasons why wheat has different cold tolerance behaviors. These results indicate that the regulation of uniquely identified DEGs are associated with varying cold tolerance.

#### Transcription Factors Related With Cold Stress

This study further examined the expression of important TFs involved in LT stress (Supplementary Table 5). In all libraries, we identified 126 DEGs annotated as TFs through the Swiss-Prot protein database. Among these, 45, 47, 4, and 58 DEGs were annotated as TFs and were detected in XMCK-XMT1, XMCK-XMT2, YNCK-YNT1, and YNCK-YNT2 comparison groups, respectively. Key TFs associated with cold stress included *AP2/ERF* (27 DEGs), *bHLH* (27 DEGs), *bZIP* (11 DEGs), *MYB* (18 DEGs), *WRKY* (8 DEGs), and *HSF*-type (8 DEGs). We found a significant difference in TFs expression under 4°C and −4°C. For instance, in the *AP2/ERF* gene family, 4 and 1 genes encoding *DREB1A* were identified and significantly expressed in YN19 and XM26, respectively, under −4°C. In contrast, the *DREB1A* gene was not identified in two cultivars under 4°C. The expression levels of 5 DEGs; *ERF79* (TRIAE_CS42_1BL_TGACv1_032766_A A0133950, TRIAE_CS42_1DL_TGACv1_061441_AA0195350), *ERF4* (TRIAE_CS42_3AL_TGACv1_194146_AA0627440), *ER F80* (TRIAE_CS42_3B_TGACv1_222710_AA0769340), *ER F73* (TRIAE_CS42_4BL_TGACv1_320475_AA1040760) were significantly higher at −4°C than 4°C in XM26. These results indicated that the increased expression levels of these genes were due to LT adaptation. In the *bZIP* gene family, *bZIP* transcription factor *RISBZ5* (TRIAE_CS42_7AL_TGACv1_556285_AA1759970, TRIAE_CS 42_7BL_TGACv1_577418_AA1875080, TRIAE_CS42_7DL_TG ACv1_603981_AA1991930) were highly expressed in XM26 than YN19 at −4°C. Similarly, in the *WRKY* family, the expression level of *WRKY46* (TRIAE_CS42_5AL_TG ACv1_375937_AA1229100) in XM26 was higher than that in YN19 at −4°C. In contrast, *WRKY70* (TRIAE _CS42_1BL_TGACv1_031026_AA0106070) is highly expressed in YN19 but not in XM26 under −4°C. Genes associated with the heat stress transcription factor (*HSF*) gene family, such as heat stress transcription factor A-2e (*HFA2E*) and heat stress transcription factor A-2c (*HFA2C*), were downregulated in YN19 at −4°C. The different expression of these TFs in two cultivars proposed that TFs may play a crucial role in response to varying LT regimes of stress in wheat.

#### Starch and Sucrose Metabolism Differently Expressed Genes Associated With Cold Stress

We further analyzed the DEGs related to starch and sucrose mechanisms (Supplementary Table 6). In this category, 36 DEGs were identified in both cultivars, and 5 of them were common genes, while 4, 14, 1, and 22 DEGs were identified in XMCK-XMT1, XMCK-XMT2, YNCK-YNT1, and YNCK-YNT2 groups, respectively. Three DEGs related to β-amylase were identified in XM26, two of which were also identified in YN19, while their expression levels were lower than XM26 at −4°C. Eight DEGs associated with sucrose synthase (SUS) were uniquely identified and upregulated in YN19. Likewise, five genes related to TPS (α, α-trehalose-phosphate synthase) and one trehalose-phosphate phosphatase (TPP) gene were uniquely identified and upregulated in XM26. Additionally, three genes (TRIAE_CS42_5DL_TGACv1_434802_AA1441860; TRIAE_CS 42_5AL_TGACv1_373994_AA1186960; TRIAE_CS42_5BL_TG ACv1_407284_AA1355280) related to TPP were uniquely identified and upregulated with high expression level while two genes (TRIAE_CS42_1DL_TGACv1_061682_ AA0201620;TRIAE_CS42_1DL_TGACv1_061682_AA0201620) associated with TPS were downregulated in YN19 at −4°C.

### Validation of Differently Expressed Genes Derived From RNA Sequencing in Cold Treated Wheat by Quantitative Real-Time PCR

**FIGURE 6:**
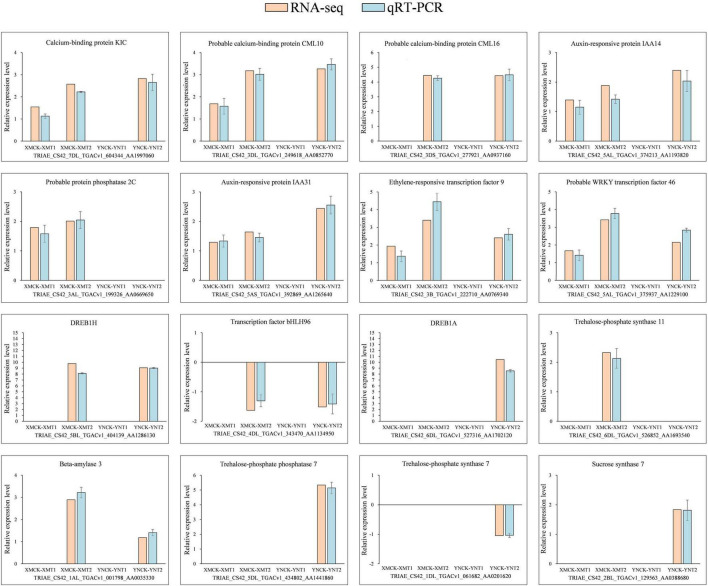
Expression validation of 16 selected DEGs. The relative expression levels of 16 DEGs were determined using qRT-PCR at 4°C and −4°C. There were 14 upregulated genes and two downregulated genes, with three biological replications and three technical replications for each. Relative expression levels were calculated by log_2_ 2^–ΔΔCt^ method and log_2_FC (RNA-seq). Here, *Y*-axis indicates relative expression levels and *X*-axis indicates each comparison group (XMCK-XMT1, XMCK-XMT2, YNCK-YNT1, YNCK-YNT2). An empty histogram indicates either RNA-seq could not detect the gene under this treatment or its expression level was low by | log_2_(foldchange)| < 1. XMCK, Xinmai26 was treated at 16°C; XMT1, Xinmai26 was treated at 4°C for 4 h; XMT2, Xinmai26 was treated at −4°C for 4 h; YNCK, Yannong19 was treated at 16°C; YNT1, Yannong19 was treated at 4°C for 4 h; YNT2, Yannong19 was treated at −4°C for 4 h.

For validation of RNA-seq accuracy, we chosen 16 differential genes related to calcium, phytohormones, and transcription factors in all groups, of which 14 were upregulated, and 2 were downregulated. As shown in Figure 6, the data from qRT-PCR for the 16 genes showed similar expression patterns to RNA-seq results, further demonstrating the accuracy of the data obtained from RNA-seq.

## Discussion

Late spring coldness has become more common in recent years due to climate anomalies in China’s wheat-growing Huang-Huai wheat growing areas of China. It becomes a primary yield-limiting factor. Therefore, the development of cold-tolerant and high-yielding cultivars is urgently needed. The previous research on LSC focuses mainly on production losses, but little relevant research has been conducted at the molecular level. Using two different cold-tolerant cultivars, this study examined how wheat spikes respond to cold stress on a physiological and transcriptional level. A comprehensive explanation of the physiological changes that occur when wheat is subjected to cold stress and the modifications in critical metabolic pathways and transcription factors could serve as a valuable basis for future studies on cold resistance mechanisms in wheat.

### Physiological Changes Associated With Low Temperature Stress

In normal growth conditions, ROS scavenging systems act as dynamic equilibrium, whereas under LT conditions, excessive ROS production disrupts the balance causing severe damage to the cell membrane and leads to lipid peroxidation. Here, the synergistic action by some protective enzymes (i.e., SOD, POD, and CAT) reduced ROS damage to plant cells (Wise and Naylor, 1987). Low temperatures stimulate several antioxidant enzymes, including SOD, POD, CAT, and others, in many plant species (Seppänen and Fagerstedt, 2000; Wu et al., 2014). The present study shown that SOD, POD, and CAT activities increased with decreasing temperature, and both cultivars exhibited significant differences for each enzymatic activity under both LT treatments (4 and −4°C) as compared to control (16°C). In addition, the comparison of two cultivars shown that the rate of antioxidant enzyme activities was higher in YN19 than XM26 in both LT and control treatments, indicating different ROS scavenging efficiencies of cold-tolerant and cold-sensitive cultivars. These findings indicated that wheat have tendency to mitigate LT damage through enhancing the enzymatic activity of antioxidant system. Basically, peroxidation of membrane lipids occurs when the rate of ROS accumulation exceeds the scavenging rate.

Due to the LT stress, higher MDA levels were observed in wheat plants. This indicates a dysfunctional dynamic balance between ROS accumulation and scavenging, the lower the temperature, the higher the degree of damage to the plant cell (Zhang et al., 2012). In this study, we found that the MDA contents of XM26 is higher than that of YN19, which indicates that the damage to the young spikes is more severe in cold-sensitive XM26 than that of cold-tolerant YN19.

Soluble sugar (SS) are considered a key osmoregulatory substance. The accumulation of SS significantly increases cytosol concentration and prevents excessive protoplasmic dehydration, which supports plant cells to avoid drought and enhance cold tolerance. Zheng et al. (2011) reported that sucrose, an integral component of SS, positively affected cold acclimation. Our results showed that SS and sucrose contents increased with decreasing temperature in young spikes. This advocates that the accumulation of SS benefit wheat plant in acclimatizing LT stress. It is worth noting that the SS and sucrose contents in YN19 were lower than that in XM26, which was consistent with the results of Zhang et al. (2019). This might be due to the increased decomposition of SS and sucrose contents in YN19 resulting from increased enzymatic activity of sucrose synthase (SUS) and sucrose phosphate synthase (SPS) (Zhang et al., 2019).

### Differently Expressed Genes Identification in Two Cultivars

We identified thousands of DEGs associated with LT in a total of four comparison groups in both cultivars, most of which showed upregulated expression, suggesting that most DEGs play an active role in wheat cold tolerance. Notably, the number of DEGs identified in YN19 was much lower than that for XM26 at 4°C, suggesting that XM26 is more sensitive to low temperature than YN19, proven in a previous study (Tian et al., 2021). Further, a greater number of DEGs were detected in response to freezing stress (−4°C) than to cold stress (4°C), indicating that a great deal of regulation occurs in response to severe cold stress. From previous studies, it has also been established that both cultivars suffer from severe LT stress and require more regulatory pathways to survive in low temperatures (Zheng et al., 2015c).

Gene ontology analysis showed number of DEGs associated with membrane (GO:0016020) and membrane part (GO:0044425), were significantly higher at −4°C, than that of 4°C, indicating that LT impacts cell membrane. Thus, these gene expressions maintain cell membrane fluidity and minimize the negative impacts of severe sub-optimal temperature. Further, many DEGs were highly enriched in the same manner (in GO analysis), such as “catalytic activity,” “binding,” and “cellular process,” indicating that different wheat genotypes have vibrant resemblances in regulating cold resistance pathways.

Kyoto Encyclopedia of Genes and Genomes analysis showed that the main enrichment pathways included plant signaling, arginine and proline metabolism, circadian rhythm-plant, and starch and sucrose metabolism. The low *q*-value for the top 20 KEGG pathways found that circadian rhythm-plant (ko04712) had a high level in four comparison groups, suggesting that circadian rhythm may considerably correlate with cold resistance. In *Arabidopsis thaliana*, circadian rhythm affects the expression of *DREB1A* through the upstream regulatory system (Artlip et al., 2013), exhibiting that the circadian rhythm is linked with cold-responsive genes. Similarly, another transcriptome analysis emphasized this argument as the LT induced circadian-rhythm pathway in *Saussurea involucrata* (Li et al., 2017). This study’s DEGs associated with circadian-rhythm were *GI* (Protein GIGANTEA), *PRR73*, *PRR95*, and *PRR1* (two-component response regulator-like PRRxx); wheat spikes may develop cold resistance because of these genes by some unknown mechanism.

### Regulation of Key Differently Expressed Genes in Hormones and Calcium Signal Transduction

Plant hormones play a vital role in combating abiotic stress by coordinating various signal transduction pathways (Peleg and Blumwald, 2011). This study noted the increased activity of several phytohormones (i.e., ABA, IAA, and ethylene) associated with the signal transduction pathway, suggesting an important role of these phytohormones in wheat response to LSC. The PP2Cs, as a negative regulator, perform an essential part in signaling under abiotic conditions (Fan et al., 2019). Three upregulated DEGs (TRIAE_CS42_3AL_TGACv1_199326_AA0669650; TRIAE_CS 42_3DL_TGACv1_249262_AA0843480; TRIAE_CS42_3B_TG ACv1_226451_AA0816990) related to PP2C were uniquely identified in XM26. Interestingly, their expressions did not differ significantly under both cold treatments. This suggests that the LT induced these PP2C genes, but their expression level may be independent of the LT level. This potential regulatory mechanism requires further investigation. Moreover, these gene expressions were not identified in YN19. These results indicate that PP2C may play a key role in cold tolerance but differs in their tolerance capacities in different cultivars.

As a plant signal molecule, ethylene plays a mediating role in abiotic stress response (Yamaguchi-Shinozaki and Shinozaki, 2006; Zhang L. et al., 2011; Peng et al., 2014). The ethylene insensitive 3/ethylene insensitive 3-like (*EIN3/EIL*) is a key component of the ethylene signal transduction pathway and has a vital regulatory role in *Arabidopsis thaliana*. Overexpressed *EIN3/EIL* plants were significantly more susceptible to freezing stress (Shi et al., 2012). In the absence of ethylene, *EIN3* is degraded by binding to EIN3-binding F-box protein 1 and 2 (*EBF1* and *EBF2*) (Potuschak et al., 2003). Previous studies have shown that increased ethylene levels are associated with reduced freezing tolerance. In *Arabidopsis thaliana*, freezing tolerance is negatively regulated by ethylene biosynthesis and signaling. It is because of cold-inducible CBFs (*CBF1-CBF3*) and type-A *ARR* (type-A *ARR5*, *ARR7*, and *ARR15*) genes are suppressed by ethylene (Shi et al., 2012; Jin et al., 2020). In this study, an upregulated gene (TRIAE_CS42_5BS_TGACv1_423203_AA1370500) annotated as *EIL2* was distinctively identified in XM26, and its expression level increased at −4°C. Whereas two downregulated genes (TRIAE_CS42_7BL_TGACv1_578130_AA1889820; TRIAE_CS 42_7AL_TGACv1_559164_AA1798420) annotated as *EBF1* (EIN3-binding F-box protein 1) were exclusively identified in YN19. We assumed that ethylene signal transduction might negatively regulate wheat’s cold tolerance.

Under optimal conditions, auxin regulates nearly every aspect of plant development; however, at times of abiotic stress like cold stress, its role is limited (Rahman, 2013). Previous studies have shown that cold stress’s consequences on auxin are linked to inhibiting intracellular trafficking of auxin efflux carriers (Shibasaki et al., 2009). Recently a new study has demonstrated that *SLR/IAA14*, as a transcriptional repressor of auxin signal transduction, plays a vital role in integrating miRNAs in auxin and cold responses (Aslam et al., 2020). In this research, 3 DEGs annotated as *IAA14* (TRIAE_CS42_5BL_TGACv1_404191_AA1289280; MSTRG.89 366; TRIAE_CS42_5AL_TGACv1_374213_AA1193820) were identified and upregulated in both cultivars, indicating that these genes are involved in wheat cold tolerance, but we still do not know the exact role of *IAA14, IAA12*, *IAA31*, and other auxin-responsive proteins in wheat response to cold stress. Therefore, whether and how auxin-regulated signal transduction affects the cold response in wheat remains to be fully discovered.

Calcium as a secondary messenger, act as a vital element in plant signal transduction in response to abiotic stress conditions (Batistič and Kudla, 2012). Calcium receptors, such as calcium-dependent protein kinases (CDPKs), calcineurin B-like proteins (CBLs), and calmodulin-like proteins (CMLs), have been well explained for their essential role in the cellular response to cold stress (Galon et al., 2010; Zhang et al., 2013). Previous study established that *CML10*, an evolutionary variant of CaM modulates the stress responses in *Arabidopsis thaliana* by regulating ascorbic acid production (Cho et al., 2016). In this study, four DEGs (TRIAE_CS42_3DL_TGACv1_249618_AA0852770; TRIAE_CS 42_3B_TGACv1_225689_AA0811070; TRIAE_CS42_3DL_TG ACv1_249184_AA0840660; TRIAE_CS42_3AL_TGACv1_19450 5_AA0634480) annotated as *CML10* were identified and upregulated in both cultivars. Interestingly, the expression levels of these genes were higher in YN19 than XM26 at −4°C. But both cultivars (YN19 and XM26) showed higher expression levels at −4°C than 4°C. We assumed that those genes might play a positive role in regulating cold resistance in wheat. We also found some genes annotated as Calcium-binding protein (*KIC, PBP1, CML16*) that were upregulated in both cultivars. Furthermore, 3 genes (MSTRG.20612; TRIAE_CS42_2BL_TGACv1_129642_AA0391380; TRIAE_ CS42_2DL_TGACv1_158699_AA0524560) annotated as calcium-dependent protein kinase 12 (CDPKC) and 2 genes (TRIAE_CS42_1BL_TGACv1_031176_AA0109230; TRIAE_CS 42_1AL_TGACv1_002163_AA0039290) annotated as calcium uniporter protein 2, mitochondrial (*MCU2*) were uniquely identified in YN19. A gene (MSTRG.26713) annotated as calcium-dependent protein kinase 3 (CDPK3) was uniquely identified and downregulated in XM26. It is assumed that these genes might be involved in Ca^2+^ signaling and are associated with cold tolerance in wheat.

### Regulation of Key Differently Expressed Genes in Transcription Factors

The regulation of transcription factors (TFs) is a primary step to maintain cell gene regulation integrity. TFs have a crucial role in plant growth, development, and resistance to biological and abiotic stresses (Wang et al., 2016). Presently, it is known that TFs (i.e., *AP2/ERF*, *NAC*, *WRKY*, *MYB*, *bZIP*, *bHLH*, etc.) are actively involved in the response mechanism against LT stress (Tweneboah and Oh, 2017). *AP2/ERF* transcription factors, especially subfamily members of *DREB* and *ERF*, play a regulatory role in a plant’s response to abiotic stress (Mizoi et al., 2012; Licausi et al., 2013). There are six *DREB1/CBF* transcription factors found in the *Arabidopsis thaliana* genome, among which *DREB1B*/*CBF1*, *DREB1C*/*CBF2*, and *DREB1A*/*CBF3* act as key molecular switches in cold response (Chinnusamy et al., 2007). In *Arabidopsis thaliana*, *DREB1A* regulates downstream gene expression by binding to the core sequence of A/GCCGAC in response to drought, high salt, and cold stress (Jia et al., 2016). The transgenic plants enhanced their cold tolerance by overexpressing *AtDREB1A* in tomatoes (Shah et al., 2016) and bottle gourd (Cho et al., 2017). Similarly, transgenic potato with *AtDREB1B* will also enhance its cold resistance (Movahedi et al., 2012). In this study, 4 genes (TRIAE_CS42_6AL_TGACv1_470976_AA1500010, log_2_FC(foldchange) = 10.47; TRIAE_CS42_6DL_TGACv1_52 7316_AA1702120, log_2_FC = 10.46; TRIAE_CS42_6DL_TGA Cv1_526864_AA1693770, log_2_FC = 10.20; TRIAE_CS42_6AL _TGACv1_472246_AA1519770, log_2_FC = 10.09) annotated as *DREB1A* were identified in YN19 with high expression level, but only one (TRIAE_CS42_5BL_TGACv1_407972_AA1360710, log_2_FC = 4.55) was found in XM26, and their expression levels were also significantly different. One gene (TRIAE _CS42_5BL_TGACv1_404139_AA1286130, log_2_FC = 9.78 in XM26, log_2_FC = 9.07 in YN19) annotated as *DREB1H* was identified at −4°C with high expression levels in both cultivars, and one *DREB1B* gene (TRIAE_CS42_5DL _TGACv1_433263_AA1407510, log_2_FC = 8.28) and two *DREB2B* genes were specifically identified in XM26. Those *DREB* genes may possibly play a key role in the cold tolerance of wheat, and it might be one of the reasons for the different levels of cold resistance between two cultivars. The sub-family members of *ERF* are involved in ethylene, jasmonic acid, ABA, and other signal transduction pathways and play a vital role in signal transduction under stressful conditions (Zhou et al., 2011). Overexpression of *ERF* transcription factor *TERF2*/*LeERF2* in tomato and tobacco promoted ethylene biosynthesis and significantly increased their cold tolerance capacity (Zhang and Huang, 2010). Moreover, *PtrERF109*, as a positive regulator, maintains strong antioxidant capacity through direct regulation of POD encoding genes by efficiently removing excessive ROS and preventing cold damage (Wang et al., 2019). In this study, two DEGs annotated as ethylene responsive transcription factor 2 (*ERF2*) (TRIAE_CS42_U_TGACv1_641158_AA2086950, log_2_FC = 2.44; TRIAE_CS42_2AL_TGACv1_094549_AA0299530, log_2_ FC = 2.23) were uniquely identified in XM26. Two DEGs annotated as *ERF109* (TRIAE_CS42_1DL_TGACv1_062736_AA 0218970, log_2_FC = 9.43; TRIAE_CS42_1AL_TGACv1_000668_A A0016720, log_2_FC = 7.58) were uniquely identified and highly expressed in YN19. This suggests that different cultivars regulate their cold resistance through different *ERF* regulation pathways. In addition, several *ERF* TFs have been identified in both cultivars, such as *ERF4*, *ERF73*, *ERF79*, and *ERF80*. These TFs may have a similar cold response mechanism in different cultivars.

In rice (*Oryza sativa*), *OsbZIP52*/*RISBZ5*, a member of the *bZIP* TFs, plays a negative regulatory role under drought conditions (Liu et al., 2012). We found that three *RISBZ5* genes were differentially expressed in two cultivars, while the expression level in XM26 was higher than YN19, indicating a negative regulatory role in response to cold stress. Other TFs, including *bHLH* (*BHLH35*, *BHLH51*, *BHLH96*, *BHLH106*, etc.), *WRKY* (*WRKY46* and *WRKY70*), and *MYB* (*MYB86* and *MYB61*), have been at least identified in one cultivar. These results indicated that the regulatory pathways of plants under LT stress were complex. Furthermore, there were differences in the regulatory mechanisms between the two cultivars. Therefore, these genes deserve further analysis in the study of cold resistance in wheat.

### Regulation of Key Differently Expressed Genes in Starch and Sucrose Metabolism

A recent report indicates that starch plays a crucial role in abiotic stresses (Thalmann and Santelia, 2017). The positive role of starch degradation for LT stress has been confirmed by many studies (Nagao et al., 2005; Sicher, 2011; Zhao et al., 2019). Beta-amylase (BAM) is an exo-hydrolase that hydrolyzes α-1,4-linked glucan chains and functions to convert starch to maltose, after which maltose can be exported from chloroplasts into the cytoplasm and then converted to glucose by disproportionating glucosyltransferases (Zeeman et al., 2004; Reinhold et al., 2011). *AtBAM3* was induced by LT stress and played an important role in its cold tolerance in *Arabidopsis thaliana* (Scheidig et al., 2002; Kaplan and Guy, 2004). In this study, 3 genes (TRIAE_CS42_1AL_TGACv1_001798_AA0035330; TRIAE_CS42_1BL_TGACv1_030979_AA0104980; TRIAE_CS 42_1DL_TGACv1_064652_AA0235250) annotated as *BAM3* were identified and upregulated in both cultivars, and the expression levels of these genes were higher in XM26 at −4°C. According to results, these DEGs were involved in facilitating the LT response and their expression patterns might be associated with the LT tolerant capacity of different cultivars. Sucrose synthesis and degradation are catalyzed by sucrose synthase (Zhang et al., 2019). In this study, six genes annotated as sucrose synthase 7 (*SUS7*) and one annotated as sucrose synthase 1 (*SUS1*) were identified and upregulated in YN19 under −4°C treatment which may play a role in the sucrose synthesis and degradation.

Trehalose, a non-reducing disaccharide with high stability and low reactivity, improves the plant’s ability to tolerate abiotic stress (Paul et al., 2008; Cai et al., 2009; Lyu et al., 2013). Some organisms have a higher tolerance to LT stress due to the accumulation of large amounts of trehalose in the tissue (Zhao et al., 2018). A recent study showed that exogenous trehalose could promote the floret fertility of wheat and alleviate the reduction of grain number per spike caused by LT stress at the booting stage (Liang et al., 2021). The metabolic pathway of trehalose formation comprises two phases. Firstly, trehalose-6-phosphate (T6P) is formed by glucose-diphosphate (UDP-glucose) and glucose-6-phosphate under the action of trehalose-6-phosphate synthase (TPS); secondly, trehalose precursor T6P is dephosphorylated by trehalose-6-phosphate phosphatase (TPP) to form trehalose, which eventually hydrolyzed into two glucose molecules (Elbein et al., 2003; Zang et al., 2011). Transgenic plants carrying TPS/TPP genes enhance their tolerance to abiotic stress by stabilizing dehydrating enzymes, protecting protein structures (Nuccio et al., 2015). Studies have shown that TPS acts as a sucrose signal of trehalose in stress response, and the *Arabidopsis* TPS gene *AtTPS2* has been proved to be a regulator of glucose, abscisic acid, and signaling under stress conditions (Avonce et al., 2004). Therefore, the DEGs encoding TPS and TPP enzymes are significant for regulating trehalose production and improving the plant’s ability to cope with abiotic stress conditions (Ilhan et al., 2015). In this study, we identified three DEGs (TRIAE_CS42_5DL_TGACv1_434802_AA14418 60, log_2_FC = 5.34; TRIAE_CS42_5AL_TGACv1_373994_AA1186 960, log_2_FC = 3.58; TRIAE_CS42_5BL_TGACv1_407284_AA135 5280, log_2_FC = 3.03) linked to TPP, they were upregulated in YN19 with high expression level at −4°C. While in XM26, 3 DEGs (TRIAE_CS42_6DL_TGACv1_526852_AA1693540, log_2_FC = 2.33; TRIAE_CS42_6BL_TGACv1_499505_AA1584 500, log_2_FC = 1.67; TRIAE_CS42_6AL_TGACv1_470862_AA149 7020, log_2_FC = 1.80) associated as TPS were upregulated and one gene (TRIAE_CS42_6AL_TGACv1_472402_AA1521780, log_2_FC = 1.77) associated to TPP were upregulated at −4°C. The results suggest that expression of the TPP and TPS genes corresponds to the response of young spikes to LTS and that YN19 might accumulates more trehalose due to high expression of the TPP genes, thus showing stronger cold tolerance. It appears that differences in the trehalose regulatory pathways of cultivars may come from their different genetic makeup and varying cold tolerance capacities.

## Conclusion

Young spikes respond to low temperatures through a number of physiological and molecular pathways. In this study, by observing a gradual temperature drop, we better understand the response patterns of young wheat spikes to natural LT conditions. Analysis of MDA content indicated that the young spikes of both cultivars were negatively affected by LSC. Meanwhile, both cultivars reduced cold damage by increasing antioxidant enzyme activity and SS content. Transcriptome analysis of XM26 and YN19 under low temperatures identified significant differences in signal transduction mechanisms, transcription factor regulation, and starch and sucrose mechanisms in two cultivars. The expression of members of the *AP2/ERF*, *HSF*, *WRKY*, *bZIP*, and *MYB* gene families were exclusively upregulated and downregulated in both cultivars. In addition, the upregulation of the sucrose synthase gene and the differential expression of TPS and TPP under LT treatments may play a key role in the cold tolerance of wheat. The present study results suggested that XM26 and YN19 have different genetic makes-up and molecular mechanisms of adaptation to cold stress; as higher ROS scavenging efficiency and increased SOD and POD contents were observed in YN19 than in XM26, attributing to their significant physiological differences. It is noteworthy that this was the first study that combined physiological and molecular attributes to understand the varying response of different cultivars to LSC. It further provides insight into the molecular mechanisms underlying cold resistance in wheat. Hence, the differential genes identified in this study may be helpful in further research in the exploration of wheat’s cold resistance mechanisms.

## Data Availability Statement

The original contributions presented in the study are publicly available. This data can be found here: National Center for Biotechnology Information (NCBI) BioProject database under accession number PRJNA770017.

## Author Contributions

GJ and MH designed the experiment and wrote the manuscript. NM and MA contributed to the data interpretation and writing. XC and YX designed the figures and formulated tables. HX, QN, BL, and WY participated in the processing of the experimental material. All authors read and approved the final manuscript.

## Conflict of Interest

The authors declare that the research was conducted in the absence of any commercial or financial relationships that could be construed as a potential conflict of interest.

## Publisher’s Note

All claims expressed in this article are solely those of the authors and do not necessarily represent those of their affiliated organizations, or those of the publisher, the editors and the reviewers. Any product that may be evaluated in this article, or claim that may be made by its manufacturer, is not guaranteed or endorsed by the publisher.

## Footnotes

1
ftp://ftp.ensemblgenomes.org/pub/plants/release-37/fasta/triticum_aestivum/dna/Triticum_aestivum.TGACv1.dna.toplevel.fa.gz

2
http://ccb.jhu.edu/software/tophat/index.shtml

3
http://bowtie-bio.sourceforge.net/bowtie2/index.shtml

4
https://ccb.jhu.edu/software/stringtie/index.shtml

5
http://www.bioconductor.org/packages/release/bioc/html/DESeq.html

6
http://www.ncbi.nlm.nih.gov/COG/

7
http://pfam.xfam.org/

8
https://ftp.ncbi.nlm.nih.gov/blast/db/

9
http://eggnog-mapper.embl.de/

10
https://ftp.ncbi.nih.gov/pub/COG/KOG/

11
http://www.genome.jp/kegg/

12
http://www.uniprot.org/

13
http://www.geneontology.org/

14
https://bioinfogp.cnb.csic.es/tools/venny/index.html

## Supplementary Material

Supplementary Figure 1 Top GO enrichment directed acyclic map of DEGs (CC: Cellular Components). **(A–D)** represents XMCK-XMT1, XMCK-XMT2, YNCK-YNT1 and YNCK-YNT2, respectively. Enrichment was carried out for each GO term and the most significant 10 terms were represented by colored boxes in each figure. It also contains the corresponding relationship of each layer. The content description and enrichment significance value of each GO term is given in each box (or ellipse). Different colors represent different enrichment significance levels, the darker the color, the higher the significance.XMCK: Xinmai26 was treated at 16°C; XMT1: Xinmai26 was treated at 4°C for 4 h. XMT2: Xinmai26 was treated at −4°C for 4 h. YNCK: Yannong19 was treated at 16°C YNT1: Yannong19 was treated at 4°C for 4 h. YNT2: Yannong19 was treated at −4°C for 4 h.

Supplementary Figure 2 Top GO enrichment directed acyclic map of DEGs (MF: Molecular Functions). **(A–D)** represents XMCK-XMT1, XMCK-XMT2, YNCK-YNT1 and YNCK-YNT2, respectively. Enrichment was carried out for each GO term and the most significant 10 terms were represented by colored boxes in each figure. It also contains the corresponding relationship of each layer. The content description and enrichment significance value of each GO term is given in each box (or ellipse). Different colors represent different enrichment significance levels, the darker the color, the higher the significance.XMCK: Xinmai26 was treated at 16°C; XMT1: Xinmai26 was treated at 4°C for 4 h. XMT2: Xinmai26 was treated at −4°C for 4 h. YNCK: Yannong19 was treated at 16°C YNT1: Yannong19 was treated at 4°C for 4 h. YNT2: Yannong19 was treated at −4°C for 4 h.

Supplementary Figure 3 Top GO enrichment directed acyclic map of DEGs (BP: Biological Process). **(A–D)** represents XMCK-XMT1, XMCK-XMT2, YNCK-YNT1 and YNCK-YNT2, respectively. Enrichment was carried out for each GO term and the most significant 10 terms were represented by colored boxes in each figure. It also contains the corresponding relationship of each layer. The content description and enrichment significance value of each GO term is given in each box (or ellipse). Different colors represent different enrichment significance levels, the darker the color, the higher the significance.XMCK: Xinmai26 was treated at 16°C; XMT1: Xinmai26 was treated at 4°C for 4 h. XMT2: Xinmai26 was treated at −4°C for 4 h. YNCK: Yannong19 was treated at 16°C YNT1: Yannong19 was treated at 4°C for 4 h. YNT2: Yannong19 was treated at −4°C for 4 h.

Supplementary Table 1 Primer sets used in qRT-PCR.

Supplementary Table 2 Pearson’s coefficients of correlation between expression levels of different samples.

Supplementary Table 3 DEGs associated with calcium signaling pathways in different groups.

Supplementary Table 4 DEGs associated with hormone signaling pathway in different groups.

Supplementary Table 5 DEGs associated with transcription factors in different groups.

Supplementary Table 6 DEGs for starch and sucrose metabolism in different groups. XMCK, Xinmai26 was treated at 16°C; XMT1, Xinmai26 was treated at 4°C for 4 h; XMT2, Xinmai26 was treated at −4°C for 4 h; YNCK, Yannong19 was treated at 16°C; YNT1, Yannong19 was treated at 4°C for 4 h; YNT2, Yannong19 was treated at −4°C for 4 h.

Supplementary Material The FPKM values of DEGs in (Sheet 1) XMCK-XMT1, (Sheet 2) XMCK-XMT2, (Sheet 3) YNCK-YNT1, (Sheet 4) YNCK-YNT2. XMCK, Xinmai26 was treated at 16°C; XMT1, Xinmai26 was treated at 4°C for 4 h; XMT2: Xinmai26 was treated at −4°C for 4 h; YNCK, Yannong19 was treated at 16°C; YNT1, Yannong19 was treated at 4°C for 4 h; YNT2, Yannong19 was treated at −4°C for 4 h.
