# Supplementary material for: Comparative Physiology and Transcriptome Analysis of Young Spikes in Response to Late Spring Coldness in Wheat (Triticum aestivum L.)
Source: Front Plant Sci. 2022 Feb 3;13:811884. doi: 10.3389/fpls.2022.811884 (PMC8850991; doi:10.3389/fpls.2022.811884)
Supplement: Supplementary Figure 3 — Top GO enrichment directed acyclic map of DEGs (BP: Biological Process). (A–D) represents XMCK-XMT1, XMCK-XMT2, YNCK-YNT1 and YNCK-YNT2, respectively. Enrichment was carried out for each GO term and the most significant 10 terms were represented by colored boxes in each figure. It also contains the corresponding relationship of each layer. The content description and enrichment significance value of each GO term is given in each box (or ellipse). Different colors represent different enrichment significance levels, the darker the color, the higher the significance.XMCK: Xinmai26 was treated at 16°C; XMT1: Xinmai26 was treated at 4°C for 4 h. XMT2: Xinmai26 was treated at −4°C for 4 h. YNCK: Yannong19 was treated at 16°C YNT1: Yannong19 was treated at 4°C for 4 h. YNT2: Yannong19 was treated at −4°C for 4 h. [file Data_Sheet_2.zip › Data Sheet 2/Supplementary Table S1.DOCX]

**Supplementary File 1: Table S1**

| Gene ID |  | Base sequence |
| --- | --- | --- |
| TRIAE_CS42_7DL_TGACv1_604344_AA1997060 | Forward | GAACAGCGTGCGACGACATT |
|  | Reverse | TTTGGGCCTTCTTCCGGTGT |
| TRIAE_CS42_3DL_TGACv1_249618_AA0852770 | Forward | TAACGCACGCACGCTCTAATGG |
|  | Reverse | CCTTCCTCGCTCCTTCCCTCTC |
| TRIAE_CS42_3DS_TGACv1_277921_AA0937160 | Forward | TGCCCAATCTCTAGCCAAGAATGC |
|  | Reverse | TATCAGTTCGGTCAGTGCGATGTTG |
| TRIAE_CS42_5AL_TGACv1_374213_AA1193820 | Forward | CAGGTTCAGCCAGCCATGTAGTAAG |
|  | Reverse | CCAGTTCCTCGGCGGTACAAAAG |
| TRIAE_CS42_3AL_TGACv1_199326_AA0669650 | Forward | GTCCCACTTCTTCGGCGTCTTC |
|  | Reverse | GCTCCTCTCCATCACCTCCTTCC |
| TRIAE_CS42_5AS_TGACv1_392869_AA1265640 | Forward | ACGGAATCCATCTACGAAGAACACG |
|  | Reverse | GCCTTGAAGAGTAGCACTGTGGTAG |
| TRIAE_CS42_3B_TGACv1_222710_AA0769340 | Forward | GCACCCGTACTTCTTCTACGAACAG |
|  | Reverse | ACAGATCAACGACCGACGAGGAG |
| TRIAE_CS42_5AL_TGACv1_375937_AA1229100 | Forward | GGAGCTTCGACGATTCTGCCTTG |
|  | Reverse | GAGATGGTGGAACTGTACGAGATGC |
| TRIAE_CS42_5BL_TGACv1_404139_AA1286130 | Forward | GCTCTCCGACTTTGCAGACATCC |
|  | Reverse | GATTGGCTACAGGTTCCGTCATCG |
| TRIAE_CS42_4DL_TGACv1_343470_AA1134950 | Forward | TCAAGAACAAGGAGGAGGTGGAGAG |
|  | Reverse | GCGTAGGATTGTGGCATGAGTGAG |
| TRIAE_CS42_6DL_TGACv1_527316_AA1702120 | Forward | ACGACACTAAGCAACACTCTCACTC |
|  | Reverse | CGTGGATGAGGAGGAGGCAGAG |
| TRIAE_CS42_6DL_TGACv1_526852_AA1693540 | Forward | GGTCATCTCCGTCTTGAATCGTCTG |
|  | Reverse | CGAACCACTTGCTCAGCTCATCC |
| TRIAE_CS42_1AL_TGACv1_001798_AA0035330 | Forward | AGCTCCATTCATTCCGTTCCGTTC |
|  | Reverse | AGCGAAGAGGAGAGGGGATTGC |
| TRIAE_CS42_5DL_TGACv1_434802_AA1441860 | Forward | TCTTCCAACCAGCAAGCGAGTTC |
|  | Reverse | GAAGTGGACGGACACGCAGAAC |
| TRIAE_CS42_1DL_TGACv1_061682_AA0201620 | Forward | TCCACAGCCCATTCCCCTCATC |
|  | Reverse | CCAGCATCCTACTACAGCAAGACAG |
| TRIAE_CS42_2BL_TGACv1_129563_AA0388680 | Forward | AAGGCTTCCTCGGCTACATCATTTG |
|  | Reverse | CAGATCGTCGGCGTGAACCTTG |
| TaActin | Forward | ACTGGGATGACATGGGGAA |
|  | Reverse | ACCGCTGGCATACAAGGAC |
